# Enhancing CAR T-Cell Function with Domains of Innate Immunity Sensors

**DOI:** 10.3390/ijms26031339

**Published:** 2025-02-05

**Authors:** Tjaša Mlakar, Mojca Skrbinek, Tina Fink, Duško Lainšček

**Affiliations:** 1 Department of Synthetic Biology and Immunology, National Institute of Chemistry, 1000 Ljubljana, Slovenia; tjasa.mlakar@ki.si (T.M.); mojca.skrbinek@ki.si (M.S.); 2 Interdisciplinary Doctoral Study of Biomedicine, Medical Faculty, University of Ljubljana, 1000 Ljubljana, Slovenia; 3 Centre for Technologies of Gene and Cell Therapy, National Institute of Chemistry, 1000 Ljubljana, Slovenia; 4 EN-FIST Centre of Excellence, 1000 Ljubljana, Slovenia

**Keywords:** CAR T-cell, cancer immunotherapy, innate immune system, Toll-like receptor domains

## Abstract

The innate immune system plays an important role in protecting the organism via recognizing the danger signals and pathogens through pattern recognition receptors. By sensing the danger signal and conveying the signaling towards the elimination of the threat, several families of these receptors, expressed on different myeloid and innate lymphoid cells, serve as the first defense line in the innate immunity. Toll-like receptors, C-type lectin receptors, and many other receptors therefore illustrate the importance of the protective role of the immune system. This was additionally confirmed by CAR T-cell-based cancer immunotherapy, where the patient’s own immune system is being used for successful tumor elimination. CAR T-cells have proven themselves to be a potent therapeutic option, yet in some cases their efficiency could be enhanced. Innate immune sensors that include strong activation and signaling domains, for instance, part of the Toll-like receptors, MyD88 (Myeloid Differentiation Primary Response gene), NKG2D (Natural killer group 2-member D), and many other domains, could be used as a CAR building module to increase the functionality and potency of the CAR T-cells.

## 1. Introduction

Cancer remains one of the leading causes of mortality worldwide. Conventional cancer therapy, which is based on surgical intervention in combination with radiation or chemotherapy, is able to target rapidly progressive proliferating cancer cells. For the past several years, biological therapies, including immunotherapy, have also become recognized as a potent therapeutic solution. The augmentation of the immune system, which plays a central role in achieving durable cancer remission, has demonstrated success, notably through the use of immune checkpoint inhibitors, which mitigate T-cell exhaustion and reinvigorate anti-tumor responses. An important advance in cancer immunotherapy occurred upon the introduction of tumor-specific T-cell receptor (TCR) engineered T-cells [1,2]. This therapy, however, had some drawbacks due to human leukocyte antigen (HLA) restrictions, therefore posing a need for allogenic cell modifications. Afamitresgene autoleucel, an FDA-approved, genetically modified, human leukocyte antigen (HLA)-restricted autologous melanoma-associated antigen 4 (MAGE-A4)-directed T-cell immunotherapy towards solid tumors, has been recently developed to overcome HLA limitations [3]. Another breakthrough in cancer immunotherapy occurred when chimeric antigen receptor T-cells (CAR T) were introduced as an MHC-independent adaptive T-cell therapy [4], making those cell products easier to generate by using principles of synthetic biology [1]. Due to the MHC-independent recognition, cancer cells that could escape the conventional T-cells by downregulating HLA and/or mutating components of the antigen presentation machinery can now be eliminated by using CAR T-cell therapy [2].

CAR T-cell therapy is a form of immune cell-based therapy that has enabled an effective and durable clinical response in the treatment of different forms of hematological cancers [3]. T-cells, which express an engineered synthetic CAR receptor, have the ability to recognize and eliminate target cells that express specific antigens through activation of the MHC receptor-independent immune response [5]. Currently, six different CAR T-based therapies are FDA and EMA approved, where two of them target B-cell Maturation Antigen (BCMA), expressed on multiple myeloma cells [6], and four of the approved CAR T-cell products are used to treat CD19+ B cell malignancies [7].

The key components of CAR receptors include an extracellular antigen-binding domain (usually a single chain variable fragment or scFv), a hinge region (usually derived from CD8, CD28, IgG1, or IgG4), a transmembrane domain (usually derived from CD3ζ, CD4, CD8α, or CD8), and one or more intracellular signaling domains (most commonly CD28 or 4-1BB) [8]. There are currently five generations of CAR receptors, which differ in the composition of the CAR receptor in order to try and overcome the limitations of CAR T-cell therapy. The 1st generation CAR encompasses only the CD3ζ domain, whereas additional activation domains (e.g., CD28, 4-1BB) are introduced into the 2nd and 3rd generations. By including additional modules (e.g., cytokine-expressing cassettes, genetic circuits that control CAR T-cell functions [9]), advanced generation of CARs emerged [10,11]. The limitations (discussed further on) that tackle this advanced CARs include antigen escape, on-off target tumor effects, tonic signaling and exhaustion of CAR T-cells, use of the cells in solid cancer immunosuppressive microenvironment etc. [12]. As stated, CAR T-cells are currently approved only for hematological cancers, as several drawbacks still exist for the treatment of solid tumors. Here, new CAR constructs carrying additional activation domains could prove themselves efficient. Therefore, many researchers are designing new CAR variants that include extra activation domains to improve the functionality and potency of CAR T-cells.

Pattern recognition receptors (PRRs) are part of the innate immune system and act as first-line defense sensors that protect organisms from infections and harmful stimuli [13]. Potent signaling molecules that are the building blocks of PRRs might not only be valuable due to their urgent role in protecting the body from bacteria and viruses but could potentially also serve as a means to boost CAR T-cell functions.

Here we overview the current state of the published CAR T-cell therapies, including different domains, originating from the innate immune system in order to potentiate the CAR-based immunotherapy, while also presenting other CAR cell-based immunotherapeutic solutions with incorporated innate immunity domains.

## 2. CAR T-Cell Therapy and Its Challenges

CAR T-cell therapies have undeniably transformed the cancer treatment landscape, especially for several hematological malignancies. However, significant challenges remain in expanding their applicability and feasibility. While the initial response rates to current second-generation CAR T-cell therapies in hematologic malignancies are remarkable, only 35–45% of patients achieve durable progression-free survival at 24 months for some cancers (e.g., diffuse large B cell lymphoma), whereas some clinical trials (e.g., for follicular lymphoma) exhibit up to 80% complete response rate [14]. This highlights the need for continued advancements to enhance the long-term efficacy and sustainability of these therapies.

Many patients who experience relapse following CAR T-cell therapy exhibit tumor cells that have undergone antigen loss, characterized by either reduced expression (antigen-low) or complete absence (antigen-negative) of the target antigen. This phenomenon allows tumor cells to evade recognition by CAR T-cells, posing a significant barrier to the long-term efficacy of these therapies [15].

Antigen escape is a significant obstacle to the long-term efficacy of CAR T-cell therapies and can arise through various mechanisms. Addressing this challenge is essential for improving patient outcomes and extending the applicability of CAR T-cell therapies. One promising approach to mitigate antigen-negative relapse is the simultaneous targeting of two surface antigens [16,17]. By incorporating dual targeting, even if one antigen is lost, the remaining target can still trigger CAR T-cell activity, ensuring tumor cell killing. This strategy is particularly viable in B-cell malignancies, which often express multiple targetable antigens, such as CD20 (Cluster of differentiation 20), CD22 (Cluster of differentiation 22) [18], or BCMA (B-cell Maturation Antigen) [19].

When the CAR T-cell persistence is insufficient or when CAR T-cells persist but adopt an exhausted phenotype, the antigen-positive relapse can occur [20,21]. Exhaustion refers to a dysfunctional state in T-cells characterized by impaired proliferation, reduced cytokine production, and diminished cytotoxic activity. This phenomenon is impaired by immunosuppressive signals, including high levels of inhibitory checkpoint molecules, such as PD-1, TIM-3, and LAG-3.

Another challenge in advancing the field of CAR T-cell therapy is the limited efficacy observed in the treatment of solid cancers. Unlike hematological malignancies, where specific antigens, such as CD19, CD20, or BCMA, are uniformly expressed and can be targeted with minimal risk of life-threatening off-target effects, solid tumors exhibit significant heterogeneity in surface antigen expression [22,23]. The efficacy of CAR T-cells in solid tumors is also significantly compromised by the immunosuppressive tumor microenvironment (TME) [12]. This poses several challenges that limit the ability of CAR T-cells to traffic to, infiltrate, and function effectively within the tumor site.

Despite improved trafficking, CAR T-cells that successfully reach the TME are subjected to a highly suppressive environment. Within the TME, various factors, such as immunosuppressive cytokines, regulatory T-cells, and myeloid-derived suppressor cells, can hinder CAR T-cell activation. These suppressive signals also accelerate T-cell exhaustion, a state of dysfunction characterized by reduced cytokine production and diminished cytotoxicity, and further limit the persistence of CAR T-cells [12]. Addressing these challenges requires the development of strategies to enhance both the persistence and functionality of CAR T-cells. This includes incorporating co-stimulatory domains within the CAR construct to promote robust expansion, improve survival, and increase resilience in the hostile tumor microenvironment.

## 3. Innate Immunity Receptors and Their Domains

During the development of the acquired immune system, the innate immune system plays a key role in combating infections through various mechanisms. Initially, physical barriers, like epithelial tissues, block pathogen entry. Antimicrobial substances, such as fatty acids, enzymes (e.g., lysozyme), gastric acid, and peptides like defensins, are secreted on these surfaces. If pathogens breach these barriers, macrophages or neutrophils detect them via receptors, leading to phagocytosis or activation of the complement system. While less specific than the acquired immune system, the innate immune system can still differentiate between foreign and self-molecules using PRRs. These evolutionarily conserved receptors identify pathogen-associated molecular patterns (PAMPs), which include DNA, RNA, bacterial components, etc. The expansion of research on PRRs receptors began after the discovery of the Toll protein in the *Drosophila melanogaster* [24,25,26,27].

There are five different classes of PRRs, where Toll-like receptors (TLRs) and inflammasomes are the most studied. Additionally, C-type lectin receptors (CLR), NOD-like receptors (NLRs), and nucleic acid sensors are known, whereas RIG-I-like receptors (RLRs) and cyclic GMP-AMP synthase (cGAS) are the most known nucleic acid sensors [25]. Activation of PRRs initiates signaling cascades resulting in proinflammatory cytokine and antiviral protein secretion, all in the manner to eliminate the pathogens. To date, domains from the TLR and CLR families have been incorporated into CAR constructs in order to augment CAR T-cell function, with their activity demonstrated in T-cells, NK cells (Natural killer cells), and macrophages (discussed further on).

TLRs are type-I transmembrane glycoproteins, characterized by the extracellular leucine-rich repeat (LRR), responsible for ligand binding and intracellular cytoplasmic Toll-IL-1 receptor (IL-1R)-resistance (TIR) homology domain required for downstream signal processing. To date, 10 TLRs are identified in the human genome (TLR1-10) and 13 in mice (TLR1-13), although TLR10 is not functional in mice. TLRs are mostly expressed by antigen-presenting cells (APCs); however, they can also be detected in other immune and non-immune cells, including T and B cells, NK cells, epithelial cells, and fibroblasts. Due to their ability to enhance signaling of APCs, which consequently promotes activation of T-cells, they represent a crucial link between the innate and adaptive immune systems [28]. The uniqueness of the TLRs is that each TLR is activated by the unique PAMP [29]. Ligand binding homo- or heterodimerization occurs, bringing the intracellular domain in close proximity and resulting in signal transduction.

TLR2 forms heterodimers with TLR1 or TLR6 to recognize bacterial lipopeptides, peptidoglycans, and fungal zymosan. TLR4 specializes in the recognition of lipopolysaccharides (LPS) from Gram-negative bacteria and endogenous ligands, such as heat shock proteins, but many other agonists are proposed for TLR4 activation. CD14 and LBP proteins are helper peptides that aid TLR4 LPS recognition and signaling [30]. Other TLRs, for instance TLR5, recognize flagellin, whereas endosomal TLRs, TLR3, TLR7/8, and TLR9 recognize single- or double-stranded nucleic acids [31,32]. Once activated, TLRs initiate signaling by recruiting adaptor proteins via interactions with the TIR domain. The TIR domain, which is present in all TLRs and their adaptors, is essential for signaling transduction. This conserved domain facilitates receptor–adaptor and adaptor–adaptor interactions through homotypic TIR-TIR binding. Following ligand recognition, dimerization of the TLRs occurs, bringing their intracellular TIR domains closer together. This structural arrangement creates a docking platform for adaptor proteins, such as MyD88 and TRIF. The TIR domain contains conserved motifs that are crucial for specificity and function. These motifs mediate the recruitment of downstream signaling molecules and determine whether the MyD88-dependent or the TRIF-dependent signaling pathway is activated. Dysregulation of TIR domain interactions can impair the immune response or contribute to pathological inflammation [33,34].

The universal adaptor is the myeloid differentiation primary response gene (88) (MyD88) protein, which, upon upstream activation, forms a multimeric signaling complex, named myddosome, that consists of six MyD88 molecules, four IL-1 receptor-associated kinase (IRAK) 4, and four IRAK1/2 molecules [35]. The MyD88-dependent signaling pathway, which is used by all TLRs except TLR3, activates IRAK and TNF receptor-associated factor 6 (TRAF6). This pathway culminates in the activation of nuclear factor kappa B (NF-κB) and mitogen-activated protein kinases (MAPKs), which promote the transcription of several pro-inflammatory cytokines, such as tumor necrosis factor-alpha (TNF-α), IL1β, and many more [36,37,38].

The adaptor proteins MyD88 and TRIF therefore mediate different TLR signaling cascades. TRIF-dependent signaling, which is specific for TLR3 and TLR4, recruits TRAF6 to activate IRF3 and IRF7. This signaling pathway induces type I interferons, which are critical for antiviral defense, and simultaneously activates TRAF6 to maintain NF-κB-mediated cytokine production. Spatial regulation of TLR signaling, plasma membrane for MyD88 and endosomes for TRIF, provides tailored responses to extracellular and intracellular pathogens [39]. The MyD88/CD40 signaling module integrates TLR-mediated innate immunity with co-stimulatory signaling. MyD88 drives NF-κB and MAPK activation and promotes cytokine production and immune cell survival. CD40, a receptor on antigen-presenting cells, recruits TRAFs upon activation, enhances cytokine production, and supports cellular activation. This module enhances the transcription of interleukin-12 (IL-12) and interferon-gamma (IFN-γ), which are crucial for robust immune responses [25,36].

The natural killer group 2-member D (NKG2D) receptor, a member of CLR, recognizes stress-induced ligands, such as MHC class I chain-related proteins A and B (MICA and MICB, respectively) and UL16-binding proteins (ULBP). In humans, it is expressed mainly on NK cells, CD8^+^ αβ (Alpha-beta)T-cells, and γδ (Gamma delta) T-cells. The activation of NKG2D relies on adaptor proteins to transduce intracellular signals. DNAX-activating protein 10 (DAP10), the primary adaptor in humans, recruits phosphatidylinositol 3-kinase (PI3K) via its Tyr-Ile-Asn-Met (YINM) signaling motif, thereby initiating the PI3K-Akt pathway. This signaling pathway regulates survival, proliferation, and cytotoxic reactions. DAP10 also participates in Grb2 and Vav1 and thus promotes the restructuring of the cytoskeleton and the motility of immune cells. In contrast, DAP12, which is mainly found in mice, uses an immunoreceptor tyrosine-based activation motif (ITAM). Upon activation, ITAM is phosphorylated and recruits the kinases Syk and ZAP70. This signaling cascade enhances cytotoxic responses and improves the ability of the immune system to eliminate stressed or transformed cells [40].

## 4. CAR T-Cells with Innate Immunity Domains

TLRs, although primarily expressed by innate immune cells, play a crucial role as costimulatory and regulatory molecules in T-cells. TLRs are pivotal in shaping T-cell responses by influencing key cellular processes. Activation of T-cells through TLR agonists has demonstrated the ability to enhance cytokine production, improve sensitivity to antigen stimulation, and foster the development of robust T-cell memory [28]. These findings underscore the promise of integrating TLR signaling into CAR T-cell design as a means to synergize innate and adaptive immune mechanisms, paving the way for next-generation immunotherapies with improved therapeutic outcomes.

**Figure 1:**
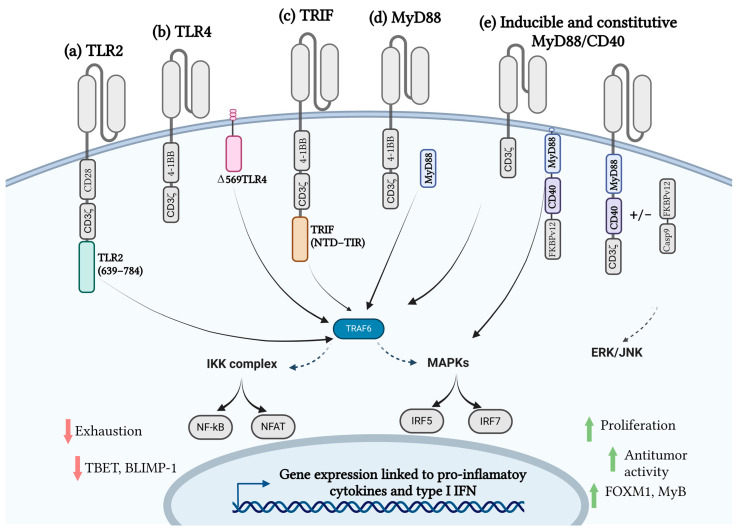
Schematic representation of CAR constructs with innate immunity signaling domains. (**a**) CAR incorporating the TLR2 domain alongside CD28 and CD3ζ (zeta) domains for activation and co-stimulation. (**b**) Membrane-bound D569-TLR4 coexpressed with a CAR containing 4-1BB and CD3ζ signaling domains. (**c**) TRIF NTD-TIR fused with a CAR containing 4-1BB and CD3ζ signaling domains. (**d**) Coexpression of MyD88 with a CAR containing 4-1BB and CD3ζ domains. (**e**) Constitutive and inducible CAR design incorporating MyD88, CD40, and CD3ζ signaling domains.

**Figure 2:**
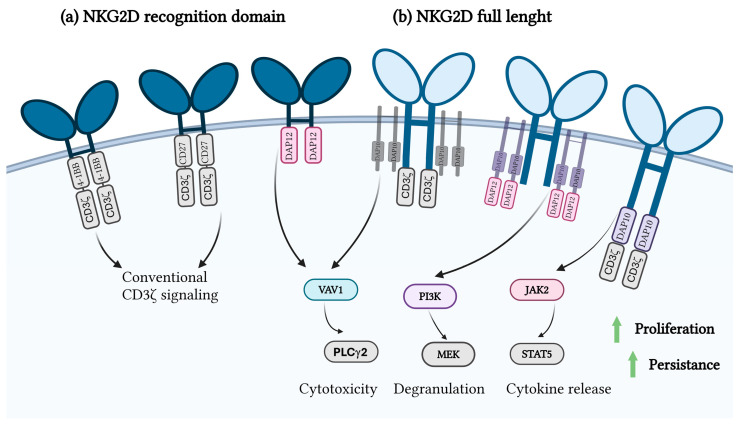
Schematic representation of NKG2D-targeting CAR constructs. (**a**) CAR construct incorporating only the extracellular recognition domains of NKG2D, enabling target-specific antigen recognition. (**b**) CAR construct incorporating the full-length NKG2D receptor, which facilitates both antigen recognition and endogenous downstream signaling through NKG2D-associated pathways.

Modifications of the receptor can consist of changes or additions to different elements of the CAR receptor. One of the possible modifications for enhancing CAR T-cell function includes the addition of different domains of innate immunity to the CAR receptor, including but not limited to TLR2 [37] (Figure 1a), TLR4 [41] (Figure 1b), TRIF [41] (Figure 1c), MyD88 [41] (Figure 1d), MyD88/CD40 [42,43] (Figure 1e), and NKG2D [44] (Figure 2), which are described in the literature so far.

### 4.1. Toll-like Receptors and CAR T-Cell Therapy

As mentioned above, TLRs play a significant role in T-cell costimulation and regulation by affecting T-cell activation, growth, differentiation, and function [45,46]. For example, it has been observed that expression of TLR in certain T-cells is associated with increased TCR signalization, resulting in elevated T-cell proliferation and cytokine production [47]. Furthermore, it has been demonstrated by Reynolds et al. that signaling via TLR2 increased proliferation and cytokine production in Th17 cells [48]. Moreover, Zhang et al. observed that mRNAs of TLR are expressed both in naïve and activated CD8+ T-cells and that activation and function of CD8+ T-cells is elevated via TLR2 stimulation [49]. In addition, stimulation of TLR leads to decreased factors associated with T-cell regulation, such as PD-1 and IL-10, consequently increasing the efficiency and antitumor effect of CD8+ cytotoxic T-cells [50]. TLR5 has also been demonstrated to serve as a costimulatory receptor of the TCR with similar effects as CD28 [51].

Incorporation of TLR into CAR constructs may represent a promising strategy to improve the constructs in terms of efficiency towards different kinds of tumors not only for hematological but also for solid tumors. Lai et al. demonstrated that the introduction of the Toll/interleukin-1 receptor domain (TIR) of TLR2 to the 3’ end of CAR constructs, targeting CD19 or mesothelin, resulted in increased antitumor responses in both solid tumors and leukemia (respectively) in vitro and in vivo. Furthermore, they have tested their construct targeting leukemia through a pilot trial in a patient with relapsed B cell acute lymphoblastic leukemia, who achieved a complete remission through treatment with CAR T-cells with added TLR2 domain. This result proves that such constructs are able to be effectively used in patients [37]. Consequently, a clinical study (NCT02822326) was conducted, where the previously mentioned findings were supported by observation that CAR T-cells with incorporated Toll/interleukin-1 receptor domain of TLR2 (i.e., 1928zT2 CAR T-cells) were able to cause complete remission in three relapsed patients with acute lymphoblastic leukemia (ALL), who were all resistant to conventional modes of treatment. During the treatment no serious adverse effects were observed, however all three patients presented with cytokine release syndrome in a lower grade, which was successfully treated by tocilizumab [52]. Moreover, since 2020 there has been an ongoing investigator-initiated phase 1 dose escalation clinical trial (i.e., ENABLE–NCT04049513) for the treatment of relapsed and refractory B-cell non-Hodgkin lymphoma (r/r B-NHL), in order to evaluate the safety and efficacy of third generation anti-CD19 CAR T-cells by incorporated signaling domains CD28 and TLR2 [53].

In addition to TLR2, incorporation of TLR4 also shows promise in terms of increased efficacy of such CAR constructs. For example, Lei et al. developed a novel second-generation M1 polarized CAR macrophage, where they introduced, in tandem, the intracellular CD3ζ domain and intracellular TIR domain of TLR4, which is able to promote and maintain the M1-like phenotype of macrophage-specific CAR (M-CAR) cells. They have observed that incorporation of the TIR domain promoted the M1-like proinflammatory macrophage state while suppressing the M2-like state, which serves as an advantage when used in a cancer immune cell therapy setting [54]. Moreover, Duan et al. demonstrated that engineering macrophages with a chimeric antigen receptor targeting VEGFR2, combined with the intracellular domain of TLR4, led to TNF-α secretion and exhibited antitumor effects in both in vitro and in vivo models [55]. Furthermore, Mikolič et al. showed that incorporation of the TLR4 domain into the 2nd generation CAR targeting CD19 resulted in increased CAR T-cell cytotoxicity and activation, while it could also lead to CD19 CAR sustainability in both hematologic malignancies and solid tumor models. They have, however, surprisingly observed that CAR TLR4 showed slower response in solid tumors in vivo; nevertheless, in all mice, full remission was achieved compared to the control conventional CAR T-cell construct [41].

Altogether these studies demonstrate a beneficial effect of the introduction of TLR or their domains into CAR constructs (Figure 1), although more studies are needed to sufficiently test the efficacy and safety profiles of such constructs.

### 4.2. MyD88/CD40 CAR T-Cell Therapy

MyD88 functions as a key adaptor protein in TLR signaling and activates IRAK kinases and TRAF6, leading to stimulation of NF-κB and MAPKs. Tumor-necrosis family member CD40, a co-stimulatory receptor, acts as an enhancer of NF-κB and MAPK activation via TRAF proteins and is thus able to increase T-cell proliferation, survival, and cytokine production [38,56,57,58]. Therefore, the integration of MyD88/CD40 domains into the CAR construct might prove beneficial in eliciting a more potent CAR T-cell response to specific cancer cells.

There are two main frameworks of MyD88/CD40 CAR T-cells currently described in the literature: first using inducible and second constitutive activation systems, each with its own advantages and limitations. Firstly, Foster et al. demonstrated that the introduction of an inducible MyD88/CD40 (iMC) molecular switch, which can be activated through the introduction of a small ligand (i.e., rimiducid) into CAR T-cells, is able to ensure a costimulatory signal that results in enhanced T-cell survival and proliferation through TCR or CAR signaling. They, however, observed that iMC on its own (without CAR signaling) is not able to drive expansion of T-cells, but it has the ability to induce a network of pro-survival cellular pathways while also ensuring necessary costimulatory signals that stimulate T-cell proliferation and anti-tumor activity. As an inducible switch, the iMC addition allows regulation of CAR T-cell function through the introduction or withdrawal of the activating ligand [59]. Furthermore, Mata et al. showed that CAR T-cells directed towards HER2 antigen, with incorporated iMC, resulted in enhanced antitumor activity, which surpasses that of the conventional CD28-HER2-directed CAR T construct [42]. Overall, both studies demonstrate that CAR constructs with iMC are able to increase the efficacy of CAR T-cells directed not only towards hematological but also towards solid cancers. To support such preclinical results, currently, there is an ongoing clinical trial where Stein et al. are assessing the safety and activity of a rimiducid-inducible MyD88/CD40 costimulation switch incorporated into CAR T-cells targeting prostate stem cell antigen (PSCA) in patients with previously treated solid tumors (NCT02744287). First results of phase 1 show that treatment with these CAR constructs resulted in preliminary evidence of biologic activity; however, toxic characteristics of CAR T-cells known from previous CAR T-cell studies were also observed [60].

Contrary to inducible MyD88/CD40, the introduction of the constitutive MyD88/CD40 construct (cMC) into CAR T-cells provides continuous signaling and thus sustained activation and cytokine production of CAR T-cells. Collinson-Pautz et al. have developed a CAR T-cell construct with incorporated MyD88 and CD40, connected via a deliberately inefficient 2A linker system, which allows continuous signaling. This approach significantly improves T-cell proliferation, survival, and anti-tumor response (i.e., production of cytokines IFN-γ, TNF-α, and IL-6), particularly directed towards CD19+ and CD123+ hematological malignancies in vitro and in vivo. The drawback of constitutive activation with cMC, however, is the increased risk of cytokine release syndrome (CRS) and other inflammatory toxicities due to excessive (toxic) cytokine secretion [43]. Contrary to iMC CAR T-cell constructs, whose activation is regulated through small molecules, cMC signaling can lead to cytokine-related toxicities. The authors have tackled this problem by the introduction of safety switches, such as inducible caspase 9 (iC9), through the implementation of selective blockade of inflammatory cytokines or with the use of CD8+ T-cells that have a tendency towards lower cytokine secretion [43].

Although iMC and iC9 both achieve robust control of MC CAR T-cell function, both are triggered by the same ligand. Furthermore, iMC single switch systems are mostly relying on a gradual weaning of the ligand. Therefore, to increase costimulation while also maintaining a good safety profile of the same CAR T-cell construct, a second regulatory switch is necessary. Duong et al. have developed a regulatable dual-switch system where they incorporated iMC costimulation and a rapamycin-induced caspase 9-based safety switch (iRC9). In this case, iMC costimulation allows for enhanced function and persistence of iMC CAR T-cells through rimiducid, while iRC9 acts as an off switch, causing rapid apoptosis and consequently termination of signaling after the introduction of rapamycin. In addition, the iRC9 has the capacity for dose-dependent sensitivity, meaning that during toxic events it allows lowering of cytokine levels and stopping the overactivation of the immune system without compromising initial CAR T-cell efficacy [61].

Moreover, Prinzing et al. have demonstrated that CAR T-cells with introduced MyD88 and CD40 signaling domains into the CAR endodomain enhanced CAR T-cell function in in vitro models during repeated stimulation and also in in vivo tumor models. In addition, they have demonstrated, using transcriptional analysis, that constructs containing MyD88 and CD40 domains have increased expression of MYB and FOXM1, which are critical for cell cycle regulation. Moreover, after stimulation, cells retained a less differentiated phenotype compared to conventional CAR, which was characterized by lower expression of markers of terminal differentiation, such as transcription factor T-bet and B lymphocyte-induced maturation protein 1 (Blimp-1) [62]. These approaches to CAR construct function enhancement can also be used in other cells of the immune system, for example, NK cells, in order to achieve an effective cancer treatment [63].

In addition to constitutive and inducible MyD88/CD40 systems, Mikolič et al. investigated the incorporation of MyD88 into CAR T-cells through expression on a separate polypeptide chain, which showed increased therapeutic potential of CAR T-cells in vitro and in vivo. This was foreseen by the authors as MyD88 signaling allows the production of proinflammatory cytokines. The potential upside of these CAR T-cell designs is the elimination of the need for chemical regulation of the MyD88/CD40 domain since the researchers did not observe any tonic signaling present in such cells [41].

Overall, these studies demonstrate that the integration of MyD88/CD40 signaling modules into CAR T-cells has redefined cellular immunotherapy by harnessing innate and adaptive immune signaling to overcome limitations, such as exhaustion, poor persistence, and limited efficacy in solid tumors. Together, MyD88 and CD40 form a dual signaling platform that significantly enhances CAR T-cell performance in preclinical models.

### 4.3. TRIF CAR T-Cell Therapy

TIR-domain-containing adaptor protein inducing IFNβ (TRIF) is able to control TLR4-induced MyD88-independent signaling, and it is in addition an exclusive adaptor used by TLR3. TRIF is also an adaptor that controls both TLR3- and TLR4-mediated type 1 IFN production [64]. Due to its role in the TLR signaling pathway, TRIF might be an interesting domain to explore in CAR-mediated signaling. Mikolič et al. developed a novel CAR construct with an incorporated TRIF domain, as they estimated that due to its function, TRIF could prove to be a beneficial addition to the CAR construct, especially for treating more complex solid tumors. They have observed that the incorporation of the TRIF domain into the CAR construct granted good results in vitro; interestingly, however, further testing in vivo revealed that the incorporation of TRIF into the CAR T-cell construct might impair CAR T-cell functionality in vivo [41]. Further studies might be needed in order to fully evaluate the benefit or lack of it in the use of the TRIF domain in CAR constructs.

### 4.4. NKG2D and CAR T-Cell Therapy

NKG2D receptor, a type II transmembrane glycoprotein, recognizes stress-induced ligands, such as MICA, MICB, and ULBPs. These ligands, which are upregulated in tumor cells due to DNA damage, oncogenic transformation, and cellular stress, are reliable markers for immune defense [40,65]. NKG2D CAR T-cells (Figure 2) uniquely integrate costimulatory and activation signaling via DAP10, eliminating the need for additional signaling domains and maintaining efficacy in targeting malignancies with heterogeneous antigen expression [66].

Preclinical studies of NKG2D CAR T-cells have shown remarkable efficacy in hematological malignancies. In AML, these cells effectively eradicated leukemic blasts, even in populations with low NKG2D ligand expression. This efficacy was enhanced by pharmacological agents, such as histone deacetylase inhibitors (HDACi), which upregulate the expression of NKG2D ligands and render AML cells more susceptible to immune attack [44,67]. It is noteworthy that the NKG2D CAR T-cells were selectively directed against AML cells, while healthy hematopoietic cells were spared, as the ligand was not present in normal tissue [68]. In T-ALL, NKG2D CAR T-cells showed robust cytotoxicity in vitro and in vivo and were resistant to immune evasion mechanisms, such as ligand shedding. However, the elimination of leukemia-initiating cells without ligand expression remains a challenge, highlighting the need for combination therapies [69,70].

The use of NKG2D CAR T-cells in solid tumors has been challenging due to immunosuppression in the tumor microenvironment (TME) and poor infiltration of CAR T-cells. Despite these obstacles, NKG2D CAR T-cells have shown significant potential. Han et al. developed a CAR construct that incorporates the extracellular domain of NKG2D as the antigen recognition component, paired with a CD3ζ activation domain and either a 4-1BB or CD27 costimulatory domain. This design enables the CAR to recognize a broad range of NKG2D ligands, leveraging the signaling cascades of the CAR construct to induce T-cell activation. The incorporation of 4-1BB or CD27 costimulatory domains significantly enhanced the anti-tumor response by promoting T-cell persistence and functionality, leading to a substantial reduction in tumor growth. However, the NKG2D CAR was unable to completely eliminate tumor cells, indicating room for improvement. While this strategy demonstrates significant potential, further optimization is needed to enhance its efficacy, including strategies to achieve complete tumor eradication and ensure robust clinical applicability [71].

In preclinical glioblastoma models, these cells efficiently crossed the blood–brain barrier, infiltrated the tumor, and significantly reduced tumor burden [44,72,73]. NKG2D CAR T-cells also effectively targeted glioblastoma stem cells, which are known for their resistance to conventional therapies [44]. In addition, NKG2D/DAP10-12 CAR T-cells containing both DAP10 and DAP12 signaling domains showed better tumor control and functional persistence compared to NKG2D-CD3ζ CARs in pancreatic, ovarian, and mesothelioma cancers. Interestingly, NKG2D/DAP10-12 CAR T-cells preferentially expanded in culture due to fratricide, driven by the stimulation of NKG2D ligand (NKG2DL) expressed by a subset of activated T-cells. These NKG2D/DAP10-12 CAR T-cells exhibited polyfunctionality, significantly reduced expression of KLRG1 and CD57, elevated CD27 levels, and predominantly effector memory differentiation. Surprisingly, the inclusion of a CD3ζ activation domain resulted in poor expansion and reduced efficacy of NKG2D-CD3ζ-CAR T-cells. Further investigation revealed that the intact CD3ζ endodomain correlated with increased NKG2DL expression, leading to diminished cell performance. However, incorporating a single ITAM-containing activation module markedly enhanced both cell expansion and tumor clearance. These findings demonstrate the importance of optimizing CAR design when targeting NKG2DL [73].

While the application of NKG2D-based CARs has shown considerable promise in targeting both hematological malignancies and solid tumors, their versatility extends beyond traditional cancer immunotherapy. One area of interest is the use of NKG2D CARs to address senescence-associated pathologies. Cellular senescence, although initially a tumor-suppressive mechanism, often accumulates in tissues over time and contributes to chronic inflammation, tissue dysfunction, and age-related diseases, including cancer relapse and progression. Senescent cells exhibit a distinct secretory phenotype and express surface ligands for NKG2D. This makes them viable targets for NKG2D CAR-mediated clearance. Unlike conventional CAR designs targeting specific tumor-associated antigens, NKG2D CARs have the ability to recognize a broader array of ligands, providing a unique opportunity to eliminate the heterogeneous populations of senescent cells that accumulate in aging tissues and pathological conditions.

Senescence-targeting CAR incorporates the extracellular domain of NKG2D and the intracellular signaling domains of 4-1BB and CD3ζ. This construct demonstrated robust efficacy in targeting and eliminating senescent cells both in vitro and in vivo. In preclinical models, including aged mice and aged nonhuman primates, the NKG2D CAR T-cells efficiently mediated the killing of senescent cells. Aged mice treated with NKG2D CAR T-cells showed significant improvements in physical performance post-treatment, suggesting that the clearance of senescent cells can contribute to functional rejuvenation [74].

Clinical studies have confirmed the potential of NKG2D CAR T-cells in both hematological and solid malignancies. A phase I clinical trial evaluating CYAD-01, a CAR T-cell therapy for the treatment of relapsed or relapsed acute myeloid leukemia (AML) and multiple myeloma (MM), demonstrated promising results. The therapy achieved significant tumor reduction while maintaining a manageable safety profile, attributed to its selective targeting of tumor cells via the NKG2D receptor. Building on the success of CYAD-01, an enhanced version, CYAD-02, has been developed. CYAD-02 retains the NKG2D receptor but incorporates a shRNA that specifically knocks down the expression of NKG2D ligands on the CAR T-cells themselves. This modification prevents self-targeting and improves the persistence and functionality of the CAR T-cells. CYAD-02 is currently being tested in clinical trials for patients with AML and myelodysplastic syndromes. In addition to the autologous therapy, the alloSHRINK study investigated the allogeneic NKG2D CAR T-cell construct named CYAD-101 in combination with chemotherapy in metastatic colorectal cancer and showed a manageable safety profile and early clinical activity [75]. In addition, a study on the administration of NKG2D CAR T-cells via infusion into the hepatic artery in metastatic colorectal cancer is currently being conducted to evaluate efficacy [76].

In order to increase the efficacy of NKG2D CAR T-cells, new strategies include the development of dual CARs that combine NKG2D with other tumor-specific antigens to reduce immune escape [77,78]. Furthermore, pharmacological stabilization of NKG2D ligands by HDACi maintains ligand expression and improves CAR T recognition and cytotoxicity [72]. Incorporation of NKG2D CAR can, in addition, be expanded to other immune cells, including NK cells [79].

## 5. Advantages and Limitations of Incorporating Innate Immune Domains in CAR T-Cells

While the integration of innate immunity domains into CAR T-cell constructs offers significant therapeutic potential, each domain has unique advantages, limitations, and mechanisms of action that need to be systematically considered. TLRs, such as TLR2 and TLR4, enhance the functionality of CAR T-cells by promoting cytokine production, improving antigen sensitivity, and enhancing cytotoxic activity [28,37,41]. These effects are largely attributed to their ability to synergize innate and adaptive immune responses [28,41]. However, the incorporation of TLR domains has shown variable results depending on tumor type, with TLR4 constructs responding more slowly in solid tumors, despite achieving remission in preclinical models [41,54,55]. The MyD88/CD40 signaling modules, whether inducible or constitutive, significantly improve the survival, persistence, and anti-tumor activity of CAR T-cells by driving the activation of NF-κB and MAPK [35,42,43,62]. Inducible designs allow external regulation and provide a safety mechanism against toxicities, such as cytokine release syndrome, while constitutive systems provide sustained signaling but carry an increased risk of excessive inflammation [43,59,60,61]. NKG2D-based CAR constructs uniquely integrate costimulatory and activation signaling without the need for additional domains, allowing them to effectively target heterogeneous antigen-expressing malignancies [40,44,65,73]. However, their efficacy in solid tumors is challenged by immunosuppressive TME [44,71,73]. TRIF domains mediating MyD88-independent signaling provide type I interferon induction and anti-tumor response but require further optimization due to their limited functionality in vivo [41,64]. Overall, these results emphasize the importance of tailoring the engagement of innate immunity domains to the specific needs of each therapeutic context, balancing improved efficacy with potential safety concerns. Future research should focus on systematically optimizing these domains to maximize their therapeutic potential while minimizing the risks.

## 6. Conclusions

Beyond conventional signaling molecules, a variety of signaling domains are being employed to enhance the safety and efficacy of CAR T-cell therapies. Among these approaches, researchers have explored a diverse range of costimulatory domains, including elements derived from the innate immune system, in order to boost CAR T-cell functionality (Table 1).

Incorporating domains from innate immune signaling into CAR T-cells holds significant potential as an innovative approach to harness the intrinsic TLR functions of T-cells. This strategy leverages the natural ability of TLRs to modulate immune responses, enabling CAR T-cells to achieve enhanced activation, persistence, and functionality. By integrating TLR signaling elements, it may be possible to improve the efficacy of CAR T-cell therapies, particularly in overcoming challenges, such as insufficient T-cell activation. Inclusion of innate immunity domains has therefore proved to be very beneficial, since several clinical trials exploring their use in CAR T-cells are already in progress. Nonetheless, attention must be given to potential risks, as the increased potency of the CAR T-cells could result in adverse effects, for example, cytokine release signaling, exhaustion, and others. In addition, the introduction of innate immunity signaling domains is not exclusive only to CAR T-cells, since NK and macrophage-based therapies also already exist. This emphasizes the potential wide applicability of such CAR designs that could be more thoroughly explored in the future. Moreover, potent CAR T-cells or other immune cells with CAR constructs could be very useful in treating solid tumors, with consideration of potential therapy drawbacks.

Incorporation of innate immunity domains into CAR constructs, however, is not the only potential mechanism of exploring different innate immunity pathways in CAR T-cell signaling. Use of agonists, which are able to activate relevant innate immunity pathways in combination with CAR T-cells, has also been demonstrated to have a beneficial effect in cancer immunotherapy. Such an approach has been, for example, used to activate the stimulator of interferon genes (STINGs) pathway [80]. It has been demonstrated that activation of the STING pathway enhances the trafficking and persistence of CAR T-cells [81], induces an endogenous T-cell response in order to overcome antigen loss [82], and leads to enhanced CAR T-cell function by inducing the secretion of cytokine IL-18 in solid tumors [83]. In addition, STING agonist has also been used to modify the TME by activation of the STING pathway, which resulted in increased efficacy of CAR T-cell therapy [84]. Moreover, it has been demonstrated by Johnson et al. that CAR T-cells can be used as a delivery system for delivering immunogenic damage-associated molecular patterns (DAMPs), which activate the RIG-I pathway, consequently increasing CAR T-cell function [85]. Moreover, future research should also focus on other targeting antigens, e.g., BCMA, CD20, CD138, etc. [86].

Exploration of innate immune signaling pathways and the use of domains of innate immunity sensors, in combination with CAR T-cell therapy, clearly shows potential for opening new avenues for optimizing CAR T-cell design to achieve more robust and durable therapeutic outcomes.

## Figures and Tables

**Table 1:** Summary of CAR constructs with incorporated domains of innate immunity.

Included Domain	Innate Immunity Sensor	Cell Type	Introduction into CAR Cell	Mode of Action	Reference
TIR domain	TLR2	T-cells	At the 3’ end of CD3ζ in a 2nd generation CAR construct, targeting CD19 or mesothelin	- Increased potency towards elimination of target cells - Increased level of cytokines IL-2, IFNγ, GM-CSF) - Upregulation of genes associated with cell adhesion, synaptic transmission and migration	[37,52,53]
MyD88/CD40–inducible	TLR	T-cells	Coexpressed with CAR construct	- Provides costimulatory signal resulting in enhanced T-cell survival and proliferation through CAR signaling - Enhanced antitumor activity	[42,59,60]
MyD88/CD40–constitutive	TLR	T-cells	At the 3’ end of CD8α TM domain, connected via deliberately inefficient 2A linker system	- Improving T-cell proliferation, survival and anti-tumor response (i.e., production of cytokines IFN-γ, TNF-α and IL-6)	[43]
MyD88/CD40	TLR	T-cells	At the 3’ end of CD8α TM domain, followed by CD3ζ	- Enhanced CAR T-cell function - Increased expression of MYB and FOXM1 - Decreased expression of markers of terminal differentiation (TBET and Blimp-1)	[62]
MyD88/CD40	TLR	T-cells	Expression on a separate polypeptide chain	- Increased therapeutic effect of CAR T-cells (production of proinflammatory cytokines)	[41]
Intracellular segment of TLR4	TLR4	T-cells	Intracellular part of a CAR construct targeting VEGFR2	- Increased secretion of TNFα - Upregulation of MHC-11, CD86 or Nos2 - Antitumor effect target cells	[55]
N terminal truncation of TLR4 ectodomain	TLR4	T-cells	At the 3’ end of CD3ζ in a 2nd generation CAR construct, connected via gt2a linker, targeting CD19	- Increased secretion of cytokines - Increased antitumor effect towards targeting cells	[41]
TRIF	TLR4	T-cells	At the 3’ end of CD3ζ in a 2nd generation CAR construct, connected via gs linker	- Increased expression of gene involved in type 1 IFN signaling	[41]
DAP10	NKG2D	T-cells	ScFv replaced with DAP10 and/or in addition to scFv region	- Immune response of T-cells through harnessing endogenous NKG2D (NK-like killing of T-cells)	[67]
NKG2D extracellular domain	NKG2D	T-cells	Paired with a CD3ζ activation domain and either a 4-1BB or CD27 costimulatory domain.	- Enables CAR to recognize wide range of NKG2D ligands, including T-cell activation - Killing of senescent cells	[71,74]
NKG2D/DAP10-12	NKG2D	T-cells	Coexpression of NKG2D with DAP10 and DAP12 endodomain	- Better tumor control and functional persistence - Reduced expression of KLRG1 and CD57, elevated CD27	[73]
NKG2D/DAP12	NKG2D	T-cells	Coexpression of NKG2D ectodomain with DAP12 endodomain	- Lower cytokine production; however, effective killing	[65]
